# “We carried her in a wheelbarrow to the clinic”: process evaluation of the AMETHIST intervention combining microplanning with self-help groups to improve HIV prevention and treatment among female sex workers in Zimbabwe

**DOI:** 10.1186/s12913-026-14137-6

**Published:** 2026-02-07

**Authors:** Fortunate Machingura, Gracious Madimutsa, Memory Makamba, Tatenda Kujeke, Albert Takaruza, Jaspar Maguma, Sanni Ali, Sungai T. Chabata, Primrose Matambanadzo, Richard Steen, Maryam Shahmanesh, Amon Mpofu, Owen Mugurungi, Andrew N. Phillips, James R. Hargreaves, Frances M. Cowan, Joanna Busza

**Affiliations:** 1 Centre for Sexual Health and HIV AIDS Research (CeSHHAR) Zimbabwe, 4 Bath Road, Harare, Zimbabwe; 2 Department of International Public Health, Liverpool School of Tropical Medicine, Pembroke Place, Liverpool, L3 5QA UK; 3 Centre for Evaluation, London School of Hygiene and Tropical Medicine, 15-17 Tavistock Place, London, WC1H 9SH UK; 4 Institute of Global Health, University College London, Mortimer Market Centre, Off Capper Street, London, WC1E 6JB UK; 5 National AIDS Council, 100 Central Avenue, Harare, Zimbabwe; 6 Directorate of AIDS and TB, Ministry of Health and Child Care, Kaguvi Building, 4th Floor Central Avenue, Harare, Zimbabwe

**Keywords:** Process evaluation, Mixed methods, Complex intervention, Zimbabwe, Female sex workers, HIV prevention and treatment

## Abstract

**Background:**

The Adapted Microplanning to Eliminate Transmission of HIV in Sex Transactions (AMETHIST) randomised controlled trial tested a combination of microplanning (peer-led risk-differentiated support) and self-help groups (SHGs) to reduce the proportion of female sex workers (FSW) at risk of acquiring or transmitting HIV infection in Zimbabwe. The trial found overall high levels of FSW engagement with treatment which further improved in intervention sites. HIV prevention uptake, however, was low and not affected by AMETHIST.

**Methods:**

We conducted a mixed methods process evaluation to assess implementation, explore FSW perceptions, and understand the trial’s outcomes. We integrated routine programme statistics, qualitative data, and an endline respondent driven sampling survey among 4444 FSW to consider fidelity of implementation, feasibility of delivery, and acceptability. We also examined whether the intervention triggered changes hypothesised in its programme theory.

**Results:**

Microplanning was successfully introduced, with peers effectively providing risk-differentiated support. Early difficulties related to mapping sex work “hotspots,”, maintaining contact with mobile FSW, and some resistance to regular risk assessments, but double the number of new FSW registered at intervention clinics compared to comparison sites (8443 v 3824), and significantly more HIV tests were performed (11882 vs. 6808). SARS-COV2 disrupted the intervention, particularly SHG. Fewer groups were established than planned, and lockdowns prevented group members meeting in person and participating in collective activities. Nonetheless, 30 of 65 established SHG remained active after two years, and more SHG members registered with clinics than those reached by microplanning alone (82 cv 76% p,0.001). Increased service use did not increase effective prevention. Over 80% FSW reported condomless sex and PrEP adherence was insufficient to achieve protection. Qualitative data show FSW prefer to “wait” until HIV seroconversion before taking daily medication. They feared conflation of ART with PrEP and resulting stigma. Concerns about side effects and immediate financial priorities undermined perceived future benefits of PrEP and condoms.

**Conclusions:**

Strengthening HIV prevention should consider how to improve FSW’s hope and agency alongside reducing stigma and supporting collective rights and action.

**Trial registration:**

The Pan African Clinical Trials Registry registered the trial 2nd July 2020 (PACTR202007818077777) which was after randomisation but before any research data were collected.

**Supplementary Information:**

The online version contains supplementary material available at 10.1186/s12913-026-14137-6.

## Background

Sex workers continue to be disproportionately affected by the global HIV pandemic, with an estimated overall prevalence of 40% among female sex workers (FSW) across Africa [1]. Persistent barriers to FSW’s engagement with health services include criminalisation and stigmatisation of sex work, their vulnerability to violence and poor mental health, and underlying poverty and gender inequalities [2]. Nonetheless, many successful examples of programmes to support FSW’s health and well-being exist, and those that are underpinned by community-led and community-based strategies to empower FSW to work collectively toward shared goals have consistently shown advantages over provision of biomedical tools or individual behaviour change messages alone [3–5].

In Zimbabwe, the Centre for Sexual Health, HIV and AIDS Research (CeSHHAR) has offered integrated clinical and psychosocial services to FSW since 2009, using a community mobilisation model. The Key Populations (KP) programme (originally called *Sisters with a Voice*) operates nationally in 86 static and mobile clinics, reaching over 30,000 FSW annually; it expanded to include male and transgender sex workers from 2018. The programme takes a rights-based approach and has increased its focus on “upstream” social determinants of FSW health over time. While clinical care remains at its core, *The KP programme* has shifted from peer education to community mobilisation and supports and trains FSW in violence response and legal case work. Tailored programmes work to reach the most neglected FSW such as adolescents and young women who sell sex and young sex workers experiencing pregnancy or struggling with early parenthood [6–8]. Trials and other evaluations of these programmes have demonstrated that a network of sex worker “friendly” clinics supported through peer-led social support can steadily increase coverage of treatment and prevention at national scale [9–11].

To build on previous work, the Adapted Microplanning to Eliminate Transmission of HIV in Sex Transactions (AMETHIST) trial tested the addition of a multi-component intervention to the regular *KP* programme. The intervention combined microplanning, a formalised peer-led outreach and referral model, with creation of self-help groups (SHG). It aimed to increase coverage of the FSW population across Zimbabwe with risk-differentiated HIV prevention and care and sought to create an enabling environment for greater FSW engagement with the services to which they were referred [12]. The ultimate goal of AMETHIST was to contribute to virtual elimination of HIV in sex transactions by strengthening FSW use of prevention and treatment thereby reducing their risk of acquisition and transmission of HIV. A cluster randomised controlled trial (RCT) was conducted across 11 intervention and 11 comparison sites with KP programme clinics. Outcomes were measured in an endline respondent driven sampling survey (RDS) administered to 4444 FSW to measure the effect of the intervention on a composite primary outcome of risk of HIV acquisition (in HIV negative women) or HIV transmission (in women living with HIV) and several secondary outcomes.

Although the trial found no significant difference between arms in the composite primary outcome, among FSW living with HIV, risk of HIV transmission was significantly lower in the intervention arm compared to the standard of care arm [13] with 93% of women living with HIV virologically suppressed. Among HIV-negative women, however, risk of HIV acquisition remained high in both groups due to low levels of consistent condom use. Furthermore, despite higher self-reported PrEP initiation and use in the intervention arm, biomarkers suggest very few of the sampled women reporting current PrEP use had protective levels of Tenofovir diphosphate. Thus, the intended outcome of the AMETHIST trial was not achieved due to lack of effect on risk of acquisition among HIV-negative FSW rather than on transmission risk among those living with HIV.

This paper reports findings from the process evaluation conducted within the AMETHIST trial to help explain its outcomes. It focuses on the strengths and weaknesses of the intervention’s implementation, how FSW perceived and engaged with each component, and implications for key behaviours and their determinants. We specifically examine the programme theory underpinning the AMETHIST intervention and seek to understand where hypothesised changes occurred as expected and through what means, and where they did not, and to what extent this was due to challenges during delivery versus flaws in our guiding logic.

## Intervention and study methods

### Intervention

**Fig. 1:**
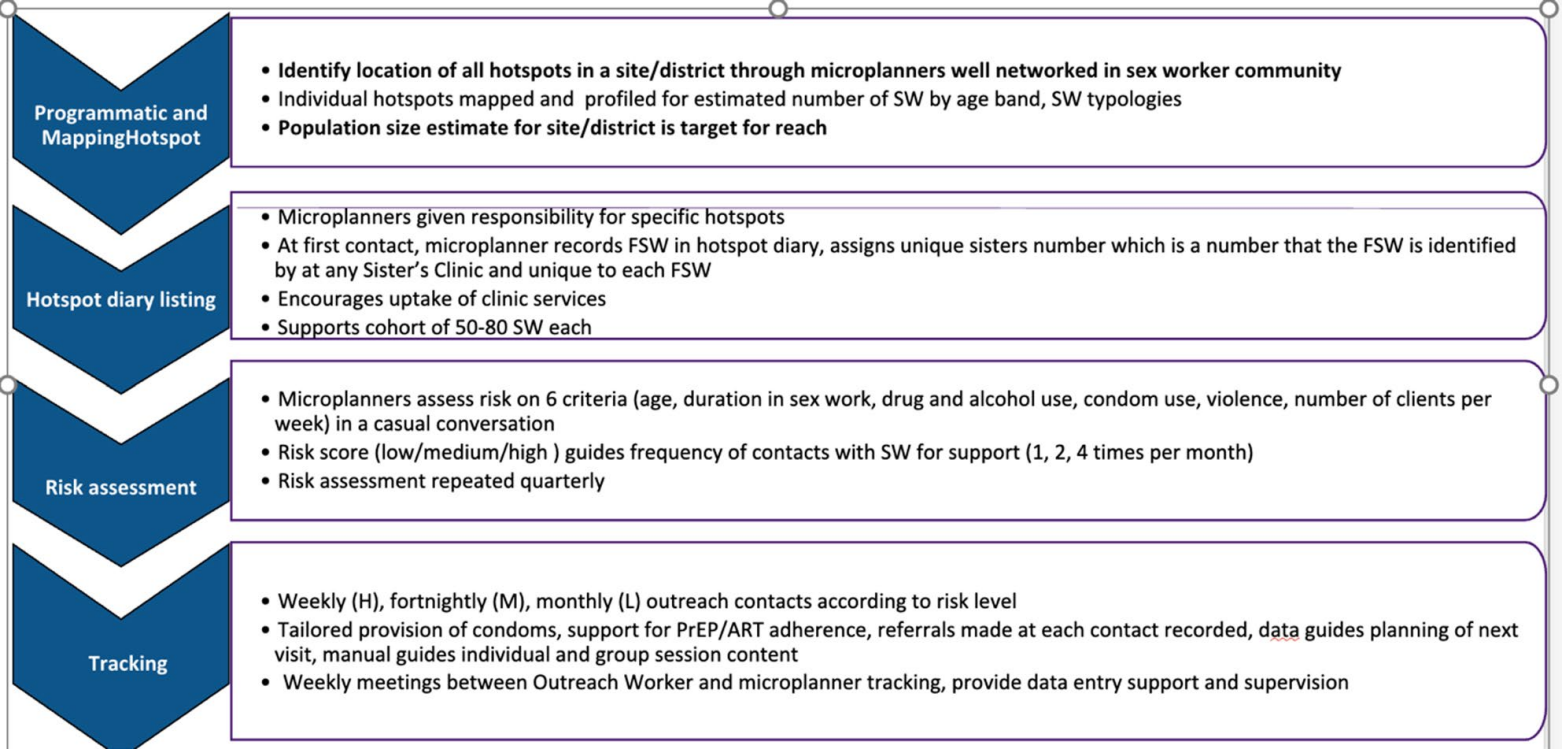
Steps in microplanning

The AMETHIST intervention grew out of the SAPPHIRE trial [10, 14] and other formative work conducted by CeSHHAR that showed gaps in coverage for the FSW population in Zimbabwe in both prevention and treatment cascades [15, 16]. To bring about a step change in service engagement and reduce risk of HIV acquisition and transmission, the intervention integrated two programmatic approaches, each based on its own theoretical rationale and evidence base. First, *microplanning* is a formalised approach to peer outreach and referral. A cadre of community peer FSW were trained to identify concentrations of sex workers, estimate their number, and take responsibility for a caseload of 50–80 individuals. Microplanners approach other FSW through community outreach, conduct risk assessments using a risk scoring tool comprising six questions on age, experience in sex work, client volume, condom use, violence and substance abuse, and provide risk-differentiated support based on individual vulnerability. FSW categorised as high-risk (score 3–5) receive weekly visits, medium risk (score 1–2) were contacted fortnightly, and low risk (score 0) at least monthly. All receive risk reduction counselling, condoms and lubricants, encouragement for regular HIV/STI testing, and support to attend services for PrEP, ART, and other relevant services i.e. for violence mitigation or substance use. This approach tailors interventions to FSW’s specific needs, maximising the impact and efficiency of HIV support. Microplanners “track” their caseload by collecting routine data on changes to the sex work environment and individual levels of risk, which they interpret during regular supervision meetings to ensure they continue to prioritise more vulnerable individuals. Figure 1 summarises the microplanning process.

Microplanning combines utilitarian concepts such as peer expertise and knowledge of “hotspot” locations with high concentration of sex workers with ideologically-driven efforts to shift ownership of health programming and its leadership to those most affected [3, 17, 18]. Prior to AMETHIST, it was successfully used in large-scale, national programmes for FSW in India and Kenya, from which our model was adapted [19, 20], although to our knowledge, AMETHIST was the first time that levels of provided support were differentiated by women’s assessed risk.

The second approach was establishment of Self Help Groups (SHG) in which FSW would come together into groups of 13–15 for mutual support, identification of shared priorities and goals, and collective action to address these. Each microplanner was expected to set up and run at least two SHG among microplanned FSWs who expressed interest in taking part, reaching an estimated 30–40% of microplanned FSW. SHG would meet fortnightly and go through a 6-month cycle of building trust and cooperation, identifying a shared project, and implementing it. Some external support was offered including occasional facilitation by an outreach worker (ORW), provision of snacks, and referrals to local organisations that could enrol group members in financial schemes such as funeral saving plans, vocational training, and internal savings and loans (ISALS). After six months, the microplanner would disengage from the group and form a second SHG to increase participation among the FSW population. The performance of SHG was evaluated based on their ability to meet fortnightly, foster trust and cooperation among members, and identify and implement shared projects for at least one year.

**Fig. 2:**
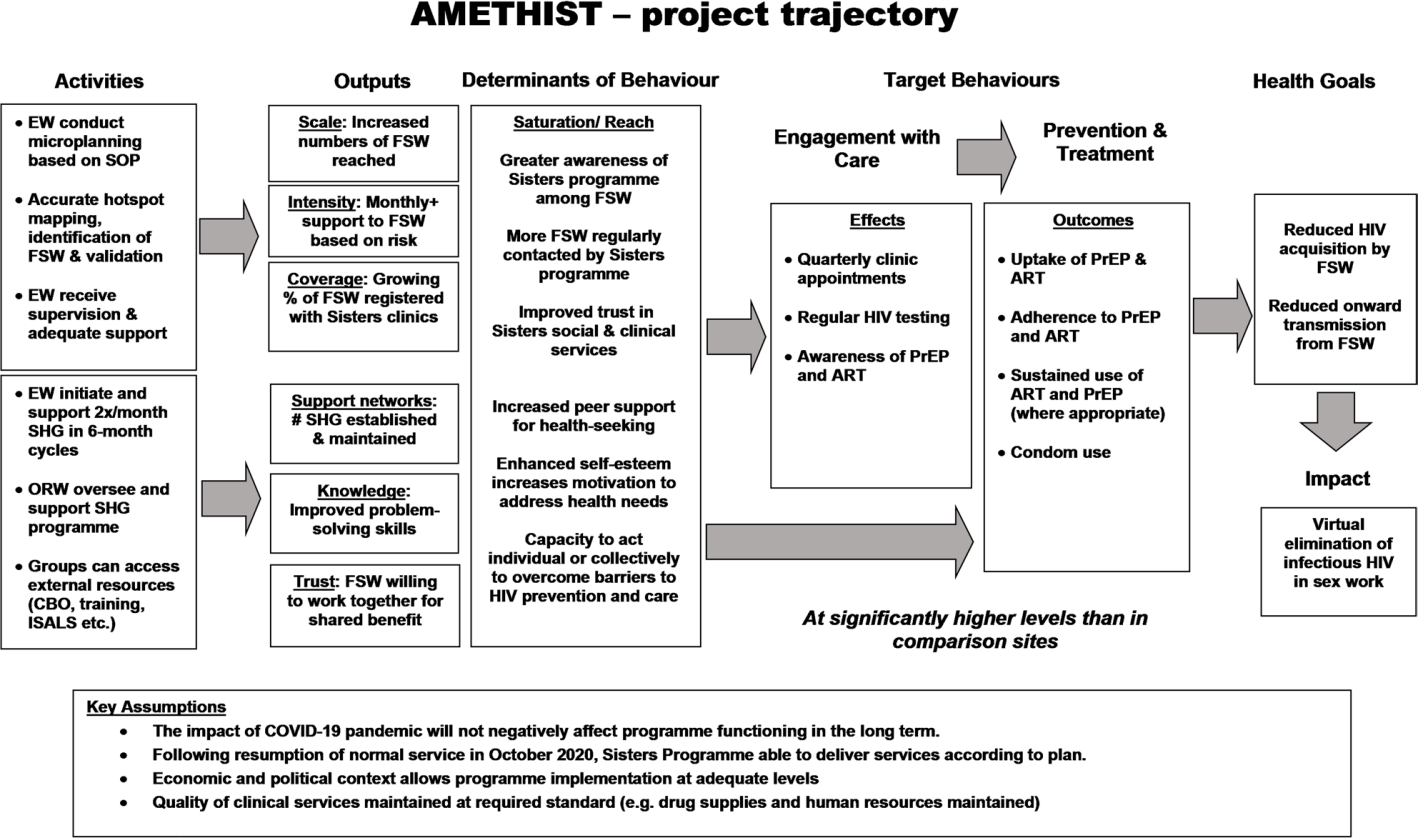
Programme logic model

SHG are part of a community development and empowerment tradition, where overcoming interpersonal conflict and going through a process of consciousness raising are seen as precursors to broader social mobilisation, particularly for marginalised and excluded populations [21, 22]. SHG have been successfully used to improve maternal and child health outcomes [23], tackle gender-based violence and improve condom use among female sex workers [24, 25]. The use of SHG is underpinned by theoretical constructs relating to social support [26, 27], primarily social cohesion (the degree of social connectedness, trust and solidarity) and social capital (norms, networks and active cooperation that enable striving for mutual benefit) [28, 29]. The exact mechanisms through which SHG lead to positive health outcomes differ across programmes and are not always made explicit [30]. Figure 2 presents the AMETHIST logic model, showing the change pathways through which the combination of microplanning and SHG sought to trigger changes in the determinants of health behaviours.

**Fig. 3:**
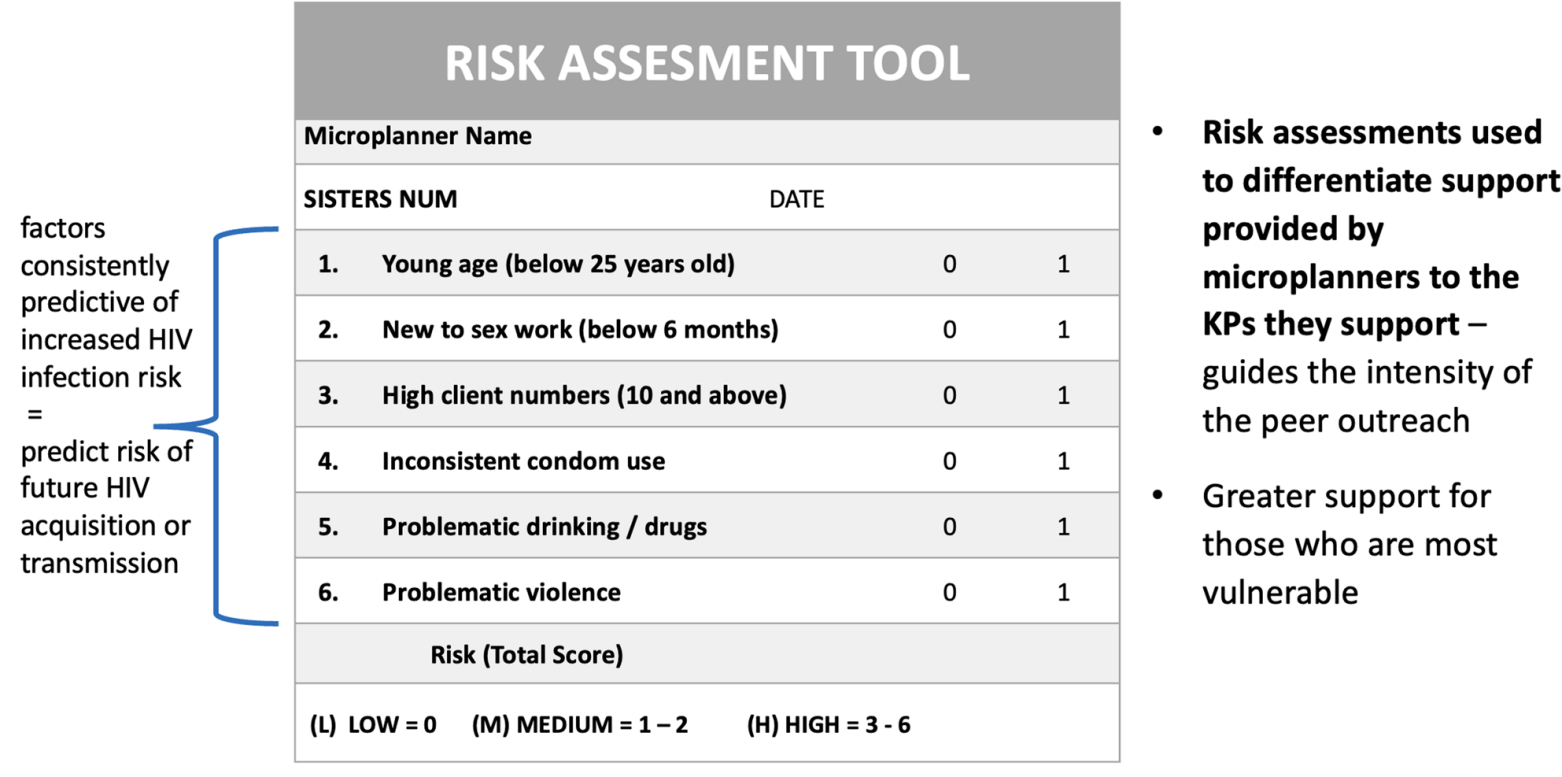
Instrument for guiding differentiated support

In the AMETHIST intervention, microplanning was introduced as a more systematic and rigorous outreach approach to the pre-existing peer education model. Microplanners, locally referred to as Empowerment Workers (EW), are FSW peer educators trained to undertake microplanning and to form and facilitate self-help groups. They sought to optimise coverage of FSW and offer risk-differentiated HIV prevention and treatment support. EW were trained to conduct detailed mapping of local hotspots by identifying specific venues and enumerating sex workers found there. Resulting population size estimates (PSE) were validated by supervising outreach workers (ORW) and repeated every six months. EW approached FSW in their assigned “hotspot” venues, conducted a rapid risk assessment, and subsequently tracked their caseload once a month, fortnightly, or weekly, depending on level of risk (see Fig. 3).

“Tracking” consisted of providing counselling, condoms and lubricants based on need, registering FSW with the *KP Programme*, and encouraging FSW to attend the clinic immediately upon registration and quarterly thereafter. FSW received regular check-ups, HIV testing followed by ART or PrEP initiation, and follow-up monitoring. Much of the role of the EW was to informally “check-in” with FSW to see how they were doing, what their concerns were, and offer general encouragement regarding uptake of services and participation in different community activities. The programme adopted a “status neutral” approach, meaning that EW were not necessarily aware of individual FSW’s HIV status.

Together, microplanning and SHG were hypothesised to increase awareness of, trust in, and motivation to engage with services above those in the standard care sites that did not have these additional social support mechanisms. Specific focus was on catalysing HIV testing and supporting initiation of and adherence to ART and PrEP. Risk differentiated support was also hypothesised to lead to increased use in interventions sites of other biobehavioural resources available at all *KP* clinics e.g., condoms and STI syndromic management, further reducing risks of HIV acquisition and transmission.

## Process evaluation

We nested a mixed methods process evaluation within the AMETHIST RCT, following an adapted version of the MRC Guidance for Process Evaluations of Complex Interventions [31] to identify domains relevant to our study, defined in Table 1, with corresponding data collection tools.

**Table 1:** Process evaluation design

Measure and Definition	Indicators and Tools	Data Produced
**Fidelity** Were intervention activities delivered as planned?	• Number of EW trained and retained in post • Number of FSW registered in EW hotspot diaries • Hotspot mapping completed every 6 months • Number of SHG established per site; number of members • Frequency of supervision visits • Perceived quality of supervision by EW	• Project records & monitoring forms • EW records of SHG delivery • Semi structured interviews with FSW & EW • Context tracker
**Feasibility** What challenges were encountered in provision of key components and were adaptations made?	• Contextual facilitators and barriers • Adaptations made to intervention design	• Semi structured interviews with ORW • Context tracker
**Coverage** Numbers and proportion of FSW reached per site for each intervention activity?	• Number and % women reached in each site per activity • Microplanning visits by differentiated risk • % microplanned women linked to care • % all FSW linked to care • Frequency of clinic attendance • Number of SHG established & maintained/dissolved • % FSW in SHG	• 4 Priority Programme Indicators (PPIs) • Respondenet Driven Sampling survey • Project records & monitoring forms
**Acceptability** Levels of participation and attitudes to/ perceptions of each stage of microplanning and SHG	• Attitudes to/acceptability of microplanning and SHG to EW and FSW peers • Participation rates (outreach, SHG, clinic registration) • Perceptions of group trust by SHG & EW	• 4 Priority Programme Indicators • Routine programme data • Semi structured interviews with EW & FSW • Focus groups with SHG members
**Quality** Did microplanning adhere to best practice standards? Were SHG sessions well conducted?	• Adherence to microplanning standards i.e. Mapping frequency; EW caseloads; EW-ORW ratios • Were SHG sessions well conducted? • Did supervision meet EW needs?	• Routine programme data • Semi structured interviews with FSW • Focus groups with SHG members
**Effectiveness** How do participants respond to intervention components? Do activities lead to intermediate steps in the change pathway?	• Changes in intended behaviour (regular service use, HIV testing, uptake of PrEP & ART) • Changes in behavioural determinants (Increased perceived support by SHG members; increased norms supporting health-seeking)	• 4 Priority Programme Indicators • Respondent driven sampling survey • Semi structured interviews with FSW • Focus groups with SHG members

## Quantitative data

Quantitative data were collected through routine programme monitoring and outreach tracking. Routine programme data were compiled into 4 Priority Programme Indicators (PPI) for microplanning. These measured the cumulative proportion of all sex workers estimated to live and work at a site registered for microplanning (intervention sites) (#1), the frequency of microplanning contacts based on FSWs’ assessed level of risk (#2), the cumulative proportion of FSW who attended the *KP* clinics in the intervention sites at least once (#3) representing the proportion of FSWs who were successfully engaged through outreach contact, and the proportion of FSW who return for quarterly visits (#4). PPI included metrics on outreach uptake by risk level, clinic attendance, and quarterly visit adherence. Outreach was measured as the proportion of FSWs reached and tracked quarterly, while clinic uptake reflected the cumulative proportion of FSWs attending *KP* clinics. Outreach frequency was calculated as the average number of monthly visits achieved for high-, medium-, and low-risk FSWs. PPI were analysed quarterly throughout the trial but more frequently for use by ORW and EW to discuss and improve progress. SHG were monitored through attendance registers and activity summary forms. The RDS survey used for the outcome evaluation included questions on exposure to intervention activities, uptake of clinical services, and perceptions of change along our hypothesised pathway i.e., increasing support between FSW and willingness to work toward shared goals. Participants were selected through a purposive sampling approach, where eight initial seeds were chosen based on specific criteria: sex work typology, engagement in services (attendees and non-attendees), and age (under or over 24 years). Each seed was given two coupons to distribute to other FSWs, inviting them to participate in the survey. Subsequently, each participating FSW received two additional coupons to recruit other peers, continuing this process until saturation was achieved. Ultimately, a total of 4,444 FSWs were enrolled in the survey. The RDS survey is described elsewhere [12].

## Qualitative data

We purposively selected 3 out of the 11 intervention sites in which to conduct in-depth qualitative data collection. These were selected based on the following criteria: one per CeSHHAR programmatic region, diversity in size of FSW population and typology of sex work, and range of programme size (e.g., number of EW employed). These sites were visited twice, within first 6 months of the intervention’s initiation, and after 12–18 months of implementation; they offer qualitative case studies on how microplanning and SHG group components interacted with each other, were responded to by the FSW community, and also highlight how local circumstances and site characteristics influenced delivery and uptake of the intervention.

1. **Chinhoyi**: Large town, roughly 1.5 hours’ drive from Harare, with a university and local mining industry, with sex work activity focused around the student population, mining camps, as well as along the highway, in bars, and entertainment venues. Northern Region (managed out of Harare). There are 2 ORW and 10 EW.
2. **Rusape**: Smaller town, roughly 2 hours’ drive from Harare, with sex work available in street, bar/entertainment, and brothel locations near a local military base. Eastern Region (managed out of Mutare). There are 2 ORW and 17 EW.
3. **Ngundu**: Largely rural, truck stop along a major highway on the way to South Africa, roughly 4 hours’ drive from Harare. Southern Region (managed out of Bulawayo). There is 1 ORW and 5 EW.

Qualitative data collection comprised semi-structured interviews and natural group discussions. At each location, 1 ORW, 5 EW and 5 FSW (including active participants, drop-outs and those who never participated) were interviewed at both time periods and discussions held with 4 SHG. Finally, 2 participatory workshops were held with 40 EWs. Trained qualitative fieldworkers conducted all interviews and group discussions, which were held in Shona, and took place at the *KP* clinic, the SHG meeting location, or, during SARS-COV2 related restrictions [32], in an outdoor space agreed by participants where privacy could be assured. Interviews were audio-recorded and transcribed into English, anonymised, and uploaded into the NVIVO software analysis package.

For this paper, we analysed the transcripts from the second round of data collection, after at least 12 months after the start of the intervention. This was to ensure enough time for respondents to reflect on later stages of implementation, to ensure we captured how earlier “teething difficulties” had been overcome and understood effects of SARS-COV2 restrictions and lockdowns.

### Ethical approval

Ethical approval was granted by the Medical Research Council of Zimbabwe (MRCZ/A/2559), London School of Hygiene and Tropical Medicine (ref:19123). and the Liverpool School of Tropical Medicine (Ref 19-115RS), the UK. Written informed consent from participants was obtained before enrolment.

## Results

**Fig. 4:**
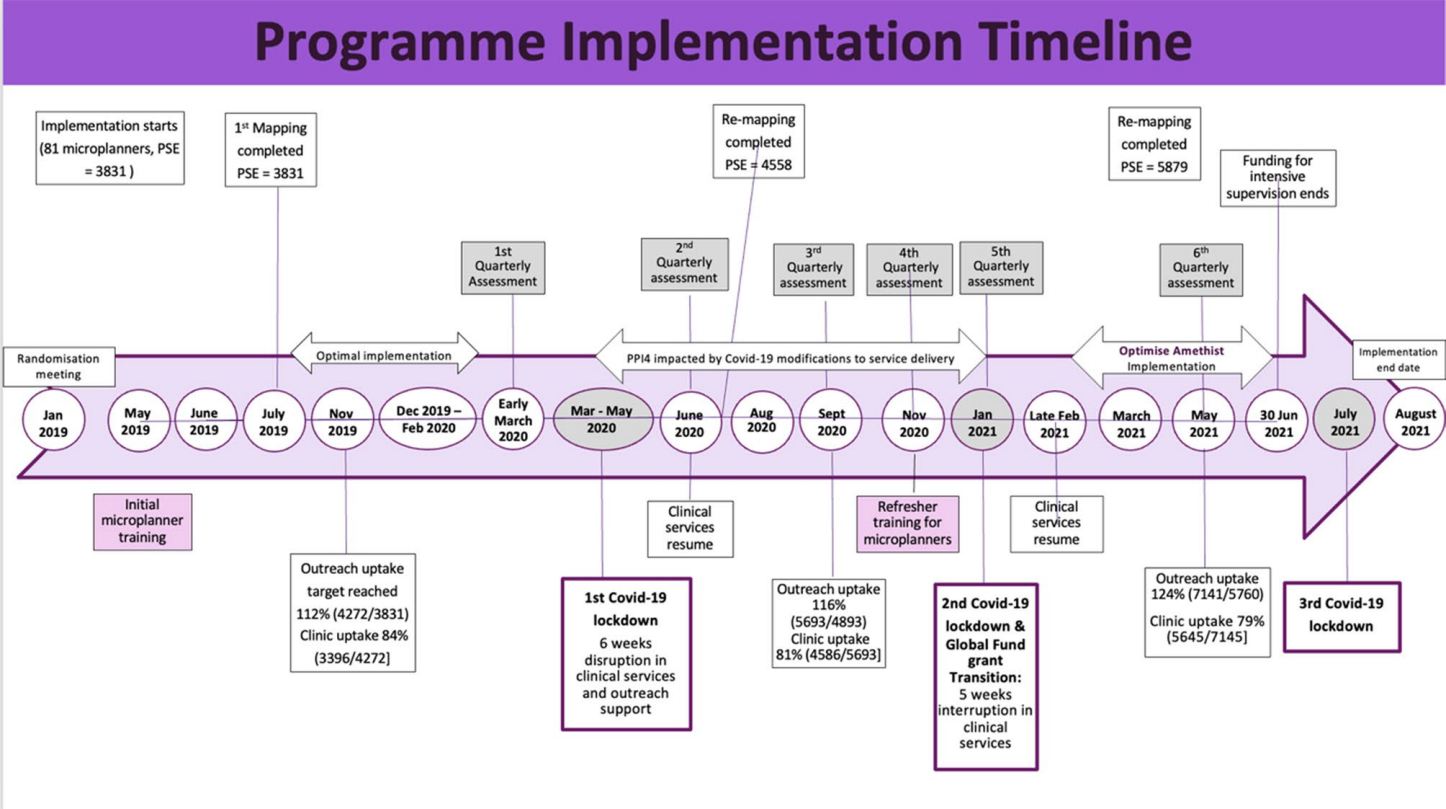
SARS-COV2 restrictions during the intervention

We first provide a narrative summary of microplanning and SHG separately, drawing on our multiple sources of data to understand strengths and weaknesses of implementation and challenges encountered. We describe where the intervention was changed or adapted and whether this was in response to observed weaknesses of the activities as designed or as a result of SARS-COV2 and related restrictions, which we consider to be anomalous and universal in terms of disrupting health services and thus adaptations as practical necessities and emergency responses rather than “course corrections”. Figure 4 illustrates the overlap of SARS-COV2 restrictions with programme implementation. Zimbabwe introduced a six-week period of complete clinic closures in 2020 followed by requirements to reduce daily client load [33]. Further restrictions included constraints on travel and in-person gatherings.

We then consider evidence for whether and how the activities delivered as an integrated package triggered expected responses as identified in the programme logic model. In this section, we attempt to understand the trial’s outcome findings by scrutinising achievements and gaps in each step across our hypothesised change pathway. Illustrative excerpts from our qualitative data are presented in Table 2 by each key component of the intervention and domain from our process evaluation framework.

### Microplanning

Microplanning was implemented as intended, although there were some early difficulties while EW familiarised themselves with the system and adjusted to having a formalised “caseload” and additional reporting forms. In total, 104 EW were recruited, 84 of whom attended the initial training and others received on-the-job training and/or attended refresher training after 1 year. EW were compensated more than peer educators ($50 vs. $15 per month) and expected to devote 20 h per week. There were some early complaints about increased burden of work and confusion about the microplanning process, which refresher training helped alleviate.

EW’s average case load was 60 FSW, within the 50–80 target, although in some sites EW microplanned more than 80, which stretched their capacity for regular tracking. The first step of the microplanning process involves mapping hotspots where sex work occurs in a given site to produce PSE. Three out of four scheduled rounds of mapping were completed in all sites, with increasing PSE over time as EW identified new or changing hotspots. However, cumulative registration of FSW for microplanning frequently achieved higher than 100% coverage of the estimated population, suggesting that either the mapping process missed eligible hotspots or individuals, or that high turnover meant some FSW listed on caseloads were no longer present or active. Table 2 presents summary statistics for key quantitative data collected during the trial.

**Table 2:** Trends in outreach contact and clinic engagement among FSWs (2019–2021)

Indicator	2019 Q2	2019 Q3	2019 Q4	2020 Q1	2020 Q2	2020 Q3	2020 Q4	2021 Q1	2021 Q2
High-risk FSWs reached weekly as intended (%)	37	55	56	68	50	63	56	58	66
Medium-risk FSWs reached fortnightly as intended (%)	58.5	68	68	82	63	77	67	63.5	74
Low-risk FSWs reached monthly as intended (%)	107	85	87	94	78	94	78	83	102
Cumulative clinic uptake (%)	95	88	84	83	83	81	81	79	79
Quarterly clinics return visits (%)	47	93	70	50	30	60	42	69	84

Outreach contact varied by risk level, with high-risk FSWs consistently reached at lower rates (37–66%) compared to medium-risk (58.5–74%) and low-risk FSWs (78–102%); however, among those who were reached, high-risk FSWs had more frequent contact with a Microplanner. Clinic uptake remained high (79–95%), but quarterly return visits fluctuated significantly, particularly during SARS-COV2 restrictions (30–93%). As shown in Fig. 4, SARS-COV2 restrictions affected outreach and clinic attendance, with a notable decline in quarterly return visits during 2020. Despite these challenges, EW achieved an average of 2.2 monthly visits for high-risk FSWs, 1.4 for medium-risk, and 0.9 for low-risk, reflecting the program’s focus on the most vulnerable (Table 3).

**Table 3:** Illustrative quotes on implementation

Domain	Microplanning	Self-Help Groups (SHGs)
Fidelity	*“In discussion*,* we would get along and understand that we are in the same trade. … I would say we will meet at the clinic*,* and I will look for your [ID] number for you*,* and we would also remind each other of clinic days.”* – **EW**,** 31 years**,** HTR**,** Rusape**	*“I will be telling them that we are organising an SHG*,* would they be interested in joining? Some will not show any interest while others will show interest. Then I set up a meeting…I told everyone that we had a meeting and those that were interested should come to this place.”* – **EW**,** 37 years**,** SHG > 5 members**,** Chinhoyi**
Feasibility	*“Some of them are hard to talk to. Sex workers have different characters. Someone might know that I’m an empowerment worker at CeSHHAR*,* if she sees me coming to her house*,* she will lock the door.”* – **EW**,** 28 years**,** LPSHG**,** Rusape**	*“The COVID situation is what is really troubling us. It’s because we are not standing together and understanding each other. We are not nicely getting together because everything is just being done online.”* – **EW**,** 35 years**,** LPSHG**,** Ngundu**
Perceived Effectiveness	*“Microplanning is more powerful than what we did as peer education and the way the girls came to the clinic - it has an impact. I liked the friendship … you can see that there is a big change.”* – **EW**,** 36 years**,** HPSHG**,** Ngundu**	*“We look for topics to educate each other about. For example*,* how to correctly wear a female condom or a male condom. We also educate each other on self-care and hygiene and also COVID-19.”* **– FSW**,** 35 years**,** HPSHG**,** Chinhoyi** *… When one of us is very sick we do accompany her to the clinic. One of us once fell sick and we carried her in a wheelbarrow to the clinic.* **FSW**,** 35 years**,** HPSHG**,** Chinhoyi**

*HTR: High tracking rate *TR: Tracked routinely *LPSHG: Low performing self-help group *HPSHG: High performing self-help group *HCW: Health care worker *LTR: Low tracking rate*LCA: Low clinic attendance*DCL: Dwindling case load *RCU: Regular clinic user

The use of a basic risk assessment tool to classify FSW into three risk categories that determined frequency of microplanning contact proved feasible. The scoring tool could be administered through casual conversation, offering EW flexibility in how they approached the process. Some FSW considered risk assessment questions invasive, judgmental, and complained the assessment was conducted too frequently. Over time, however, EW developed trust with most FSW whom they tracked, but challenges related to frequent FSW mobility remained. Overall, including periods when outreach was curtailed by pandemic restrictions, EW achieved an average of 2.2 monthly visits out of an intended 4 for the most vulnerable (high-risk) FSW, 1.4 out of 2 for medium-risk, and 0.9 out of 1 for low-risk.

PPIs show that EW were good at locating FSW and initiating contact with them, although it proved harder to ensure registered FSW attended routine clinic visits, which were recommended on a quarterly basis. However, routine clinic visits were suspended between September 2020 and June 2021 as a result of SARS-COV2 restrictions if FSW were asymptomatic and had no medication to collect. Nonetheless, 83% of FSW listed on EW caseloads ever attended a *KP* clinic and intervention clinics registered over twice as many new FSW than those in control sites over the trial period (8443 v 3824) and administered many more HIV tests (11882 vs. 6808). Clinic data also show higher uptake of PrEP and ART in the intervention sites. Microplanning appeared to increase the frequency and coverage of clinic visits across the FSW population as intended. Among the RDS sample, 68.1% reported that they had ever visited a *KP* clinic and those who also reported being microplanned were significantly more likely to report having ever visited compared to those who did not (90.5% v 39.4% *p* < 0.001). When asked about clinic attendance in the previous 12 months, again those who reported having been microplanned were significantly more likely to have attended (79.6% v 28.9% *p* < 0.001).

Microplanning as a monitoring tool proved useful for identifying implementation problems, identifying appropriate solutions and testing their effectiveness. For example, restrictions on travel during SARS-COV2 lockdowns interrupted face-to-face outreach and caused clinic closures on three occasions while other, shorter interruptions occurred due to funding delays, lack of petrol or phone credit. In all of these, the extent of the disruption could be tracked with PPIs, which also provided evidence of progress once solutions were introduced such as replacing in-person meetings with phone-based outreach and simplifying ART and PrEP prescription refill procedures. Making service provision more community-based led to considerable increases in PrEP initiations, as previously reported [34]. ). Thus the assumptions we included in our logic model about ability of both microplanning and clinic services to function after a short disruption were borne out. Services did not experience political or economic problems although all of Zimbabwe experience a short period of PrEP stock outs in 2021.

## Self help groups

The 104 EW founded 65 SHG, with a total of 1361 participants. After 28 months, 30 SHG remained active, with varying degrees of intensity in terms of regular meetings and/or attendance of members. The RDS data show that among all surveyed FSW, 26.1% in intervention sites reported belonging to a group for mutual benefit, compared to 20.9% in control arms (*p* < 0.001). In addition to meeting regularly for discussions and sharing problems, some SHG tried *mukando*, a traditional saving system where members take turns receiving an agreed amount from the others on a weekly or monthly basis, while others pooled money toward the purchase of bulk goods for resale or to distribute among themselves (i.e., taking advantage of wholesale prices for large quantities of cooking supplies or household goods).

Implementation of SHG faced the greatest challenges within the programme because SARS-COV2 restrictions not only made it difficult for group members to meet in person, but also altered the sex work environment. Qualitative data show that as FSW lost business and therefore income, they were unable to contribute to saving schemes or investments. While some groups tried to maintain momentum through remote means (e.g., WhatsApp groups), they felt frustrated at the inability to address their economic difficulties at the time of greatest hardship and their inability to meet in person reduced their ability to form bonding social capital and strengthen trusting relationships. Our logic model assumptions about the ephemeral effects of SARS-COV2 thus did not hold in relation to SHG.

Nonetheless, women who were both microplanned and joined SHG had slightly higher rates of clinic registration than those who were microplanned alone (82.7% vs. 76.1% *p* < 0.001). Group members discussed health issues during meetings, actively encouraged FSW to attend the clinics, and accompanied one another to test for HIV, collect medication or seek STI treatment. However, selection of SHG members depended on their willingness to join as well as EW’s recruitment approach. EW identified differences between those interested in building relationships and those who felt friendships between FSW were impossible or undesirable, suggesting coverage was likely to be selective.

## Effects on risk behaviour

Although AMETHIST did not significantly reduce risk of a combined measure of HIV acquisition or transmission among the FSW population, this was due to a lack of effect on prevention of acquisition. Engagement across the care cascade did improve among FSW living with HIV. For HIV-negative FSW, we hypothesised that risk-differentiated provision of condoms and increased engagement with KP services would lead to more condom use, HIV testing and PrEP use. While risk-differentiated support by EW occurred, this did not translate into preventive behaviour. Only 20% FSW reported no condomless sex in the preceding month. This corroborates data from risk assessments conducted by EW, where inconsistent condom use was reported by close to half of all FSW and was considerably higher among those with problematic drug or alcohol use (72%), or who had more than 10 clients per week (67%) or reported problems with violence (66%). FSW struggle to use condoms despite good access to them, including community-based delivery, even during SARS-COV2 lockdowns.

Similarly, our PrEP use data show that while there was an impressive number of PrEP initiations in AMETHIST intervention sites (3377 vs. 1610), the adherence necessary for achieving protection did not occur. Among 491 self-reported current PrEP users who underwent blood testing, just 2 had protective plasma Tenofovir-diphosphate levels (> 700fmol/punch) with 38 having levels 350–700 fmol/punch. Qualitative data show that scepticism, misconceptions, and reluctance to maintain PrEP use negatively affected use. FSW did not want to be mistakenly identified as taking ART and face HIV stigma and discrimination, there was widespread belief that it was better to “wait” until HIV seroconversion before taking routine medication, and rumours circulated that once on PrEP, any interruption of adherence would increase susceptibility to HIV infection.

*People say they look like ARVs. … My boyfriend came over and went straight for the place I keep my pills and asked if I take HIV medication. I told him to go and ask [nurse] what the pills are for because she works at the clinic. The issue ended when we went to [nurse] and when I also went to the clinic to get self-test kits to test myself so he could see that I am not positive.* ***FSW***, **22 years**,** LCA**,** Chinhoyi**

*… Some will tell you that PrEP is similar to ART. They can tell you that ART is taken daily*,* and we now want them to take a tablet daily. So*,* what’s the difference… they might as well wait until they are HIV positive since they are sex workers?* **EW**,** 23 years**,** LPSHG**,** Chinhoyi.**

*Some had said if you are given PrEP and you forget to take it for a day or two and the condom bursts you will be at high risk of contracting HIV. So*,* the best way is to stay as you are without taking PrEP and wait until you get HIV.* **FSW**,** 42 years**,** TR**,** Rusape**

On the other hand, uptake and sustained use of ART was high, and while HIV stigma posed a barrier to PrEP use, it was less likely to deter HIV-positive FSW from treatment. Interviews with FSW on ART provide evidence of the normalisation of treatment and acceptance of HIV as a manageable chronic condition (potentially less threatening than other common illnesses).

*You can actually hear people say that these days*,* HIV is actually better than sugar diabetes. If you are still able to take your tablets*,* you should just take them. I don’t see a problem of stigma and discrimination. AIDS is no longer scary because there are pills now and it’s all good.* **FSW**,** 30 years**,** LCA**,** Rusape**

Thus, the improved contact and service use that microplanning and SHG facilitated sustained longer-term engagement among women living with HIV compared to those in need of prevention.

## Discussion

The AMETHIST intervention combined microplanning and SHG as key ingredients to reach FSW with risk-differentiated and enhanced social support. It proved feasible to deliver as intended, although there were gaps in fidelity related to incomplete “hotspot” mapping, particularly at the beginning as EW became familiar with the process and conducting regular risk assessments and following these up with appropriate tracking frequency. As with many programmes for key populations [35–37], SARS-COV2 disrupted delivery, most seriously the implementation of SHG, which relied on regular group meetings and social interactions. The programme was able to realign most activities, for example by providing more community based and remote service options, including ART and PrEP provision. Indeed, introduction of these new measures appeared to increase PrEP uptake, although this proved an ephemeral effect [34]. While some SHG tried to remain functioning through phone-based communication, they were considerably weakened by restrictions and by SARS-COV2’s negative effects on sex work more generally, making it less likely that FSW would earn enough money to put toward savings or financial ventures. Sex workers’ already precarious economic position worsened during the pandemic in many settings, and they did not benefit from measures provided to alleviate loss of earnings for workers in less marginalised occupations [38–40].

As specified in the programme logic model, microplanning’s systematic approach to identifying, enumerating, and engaging FSW based on levels of risk did improve numbers of FSW attending regular appointments at the clinic, testing for HIV, and initiating ART and PrEP. Microplanning has already been successfully introduced elsewhere, including in Kenya [20]. Over time, it is hoped microplanning will further increase FSW ownership of the *KP Programme* through enhanced capability to analyse data and tailor activities accordingly. EW should eventually move into supervisory roles as in India [4]. There is some evidence that SHG increased members’ identification of shared identity and priorities, strengthening their capacity to work collectively (i.e. in shared business schemes) and to support and aid individuals (i.e. offering financial assistance in a crisis; taking a sick FSW to the clinic). The higher clinic attendance among SHG members might indicate that groups fostered proactive health-seeking norms and reduced anxieties about potential stigmatisation, offering both emotional and instrumental help (e.g. accompanying others), as other models of group-based support have shown [30, 41].

Despite increased engagement with services, preventive behaviours did not significantly differ between trial arms. There appears to be a specific form of stigma around PrEP, such that FSW feel it is better to avoid it and wait until treatment becomes necessary. Many FSW expressed the fatalistic belief that all sex workers eventually contract HIV and displayed lack of optimism or a sense of agency about successful prevention. They also feared prevailing conflation of PrEP with ART (both in terms of appearance, but also the view that the drug is basically the same) and did not want others to assume they were already living with HIV. Other studies have found similar slow uptake, including where reported adherence proved inaccurate once drug levels were measured [42]. It could be that it is still “early days” for PrEP use; previous work showed that while there was a core of “early adopters”, PrEP was not gaining acceptance as quickly as anticipated [16]. This is an area that requires significant more exploration particularly as it has implications for future CAB-LA, which would overcome some but not all identified barriers [43].

FSW’s positive perceptions of the quality of *KP* services in Zimbabwe has been consistently documented [9, 14, 44], and reflected in qualitative data in this study, alongside complaints and frustrations when there have been delays and gaps in coverage due to SARS-COV2, funding constraints, logistical challenges. The improved engagement with the care cascade among FSW living with HIV is further evidence of trust in the programme and services to which it refers FSW. HIV treatment has increased in acceptability and our previous work showed that for many FSW, perceived stigma of selling sex can be higher than that of living with HIV [45]. The seemingly intractable challenges of prevention among this vulnerable group may reflect pervasive sex work stigma, and also increasing desperation to prioritise current financial need over longer-term health benefits at a time of globally rising costs. It is also possible that the dynamics of sex work are changing, as they have in the past, particularly during times of economic instability [46]. If sex work is becoming more diffuse and less formalised it may be that women selling sex are less likely to identify as FSW and attend a service associated with sex work. Prior research has identified this as an issue, particularly among younger FSW and those who are newly selling sex, both attributes that would put women at high risk of seroconversion and are associated with lower preventive behaviours [47–50].

## Conclusions

The AMETHIST intervention successfully combined microplanning and SHG to engage FSW with risk-differentiated social support, enhancing access to HIV prevention and treatment services. Delivery was feasible, but SARS-COV2 disruptions had an impact. FSW positively responded to integrated-microplanning and SHG in ways aligned with our intervention’s logic model, namely that ongoing, formalised and risk-differentiated peer support helped identify and track the most vulnerable FSW, encouraging them to work together to overcome problems and increase confidence in using targeted services. We also attempted to shed insights into the discrepancy in effects on treatment and prevention outcomes found in the trial; feelings of fatalism, anticipated stigma, and conflation of PrEP with ART persisted as barriers to prevention, limiting uptake despite increased availability. The study underscores the need to better understand barriers to prevention and continue to seek ways to overcome these. Strengthening HIV prevention should consider how to improve FSW’s hope and agency alongside reducing stigma and supporting collective rights and action. This is likely to necessitate renewed focus on FSW’s mental health as well as targeted messaging about the advantages of prevention despite the increasing reality of HIV as a manageable chronic condition.

## Supplementary Information

Below is the link to the electronic supplementary material.

Supplementary Material 1

## Data Availability

No datasets were generated or analysed during the current study.

## Abbreviations

AMETHIST: Adapted Microplanning to Eliminate Transmission of HIV in Sex Transactions
ART: Antiretroviral therapy
CeSHHAR: Centre for Sexual Health, HIV and AIDS Research Zimbabwe
EW: Empowerment worker
FSW: Female sex worker
KP: Key population
HIV: Human immunodeficiency virus
ORW: Outreach worker
PPI: Priority Programme Indicators
PrEP: Pre-exposure prophylaxis
PSE: Population Size Estimate
SARS-COV2: Severe acute respiratory syndrome coronavirus 2
SHG: Self-help group
