# Supplementary material for: “We carried her in a wheelbarrow to the clinic”: process evaluation of the AMETHIST intervention combining microplanning with self-help groups to improve HIV prevention and treatment among female sex workers in Zimbabwe
Source: BMC Health Serv Res. 2026 Feb 7;26:334. doi: 10.1186/s12913-026-14137-6 (PMC12967012; doi:10.1186/s12913-026-14137-6)
Supplement: Supplementary file 1 — Supplementary Material 1 [file 12913_2026_14137_MOESM1_ESM.doc]

**Additional file 1**

Additional file 1: **Appendix A:** AMETHIST Trial: Topic Guide for FSW in Intervention Sites. **Appendix B:** AMETHIST Trial: Topic Guide for Empowerment Workers in Intervention Sites. **Appendix C:** AMETHIST Trial: Discussion Guide for SHG in Intervention

**Appendix A: AMETHIST Trial: Topic Guide for FSW in Intervention Sites**

During this interview, the aim is to understand how local sex workers perceive activities conducted as part of the AMETHIST trial, examining their awareness, use and perceptions of microplanning, Self Help Groups, clinical services, and uptake of HIV testing and PrEP or ART. These interviews also explore the current social environment for sex workers, including their relationship with each other and the wider community, and to what extent they feel sex workers can support one another and work together for mutual benefit.

# Part I: Background Information

1. **Can you tell me a little bit about yourself and your work?**

- How long have you been living and working in this area?
- Whom do you live with around here?
- Where do you work? Please describe your pattern of sex work, including where you find clients and how often you sell sex?

1. **In this study, we are interested in what life is like for sex workers around here**

- Can you tell me about the challenges sex workers face?
- What are the positive aspects of being a sex worker here?
- What do you personally find most difficult?
- How do you cope with your difficulties? [**Probe**: whom can you talk to about your problems? Whom can you ask for help?]
- How do people in the community treat you? [**Probe**: Local residents? Police? Health workers? Others?]

# Part II: Relationships between sex workers

1. **I would now like to ask you about how sex workers interact with each other. In your experience, how are relationships between the sex workers whom you know?**

- What different kinds of sex workers are there locally? [**PROBE**: Where do other women sell sex? Do other sex workers have different types of clients?]
- How do different kinds of sex workers get along?
- Can you describe friendships with other sex workers? [Probe: do you have some close friends whom you work with? Why or why not?]
- Can you give examples from work about how sex workers help each other out? [**Probe**: Please give any personal examples you remember.]
- What about outside of work, do you socialise with other sex workers?
- Can you give examples of how sex workers support each other out outside of work?
  [**Probe**: Please give any personal examples you remember.]
- What about bad experiences? Can you describe any situations where other sex workers have caused problems for yourself or others whom you know? [**Probe**: Please give some examples.]
- Have relations among sex workers changed at all since you’ve been working here? Please describe any changes.

# Part III: Sisters with a Voice

1. **You may have heard about the Sisters with a Voice programme. Can you describe it to me?**

- Can you tell me what you know about the Sisters programme?
- What kinds services do they offer?
- Can you tell me how often and where Sisters activities and services occur?

1. **Have you yourself ever participated in any Sisters with a Voice activities or used their services?**

**IF YES : *Please describe your involvement with Sisters with a Voice, including all the different activities or services you’ve used? [Let respondent fully answer before probing]***

**PROBES:**

- Is there an *Empowerment Worker* who contacts you? If so, please describe how often you see her, and what you talk about.
  - **If YES**: What do you think about these meetings? How do you benefit? Are there any things you don’t like about being contacted by the Empowerment Worker?
  - **If NO**: Have you heard about Empowerment Workers who contact sex workers around here and provide information and advice to sex workers? What do you think about them?
- Have you ever gone to the *Sisters clinic*?
  - **If YES**: Tell me about your experiences at the Sisters clinic. How do you feel about the treatment you get there? What are good and bad things about the clinic and the services there? How does the Sisters clinic compare to other health services around here?
  - **If NO**: Do you know about the Sisters clinic, which is a special service that come to the xxx health centre ever xxx weeks to provide medical services and advice to sex workers? What do you think about it? Can you explain why you have never attended?
- Can you tell me about *Self-Help Groups* organised by the Sisters Programme? [**Probe**: What have you heard about these groups? Do you think they are useful for sex workers? Why or why not?]
  - Are you part of a SHG? If so, please describe what you do in the group.
  - Do you think you benefit from your participation? How? [Or Why not?]
  - What are the challenges of being part of a group?
- Have you attended any *Community Mobilisation* sessions?
  - Why did you decide to attend/ not attend?
  - **If YES**: How many times and what do you think about them?
  - What do you think is the most useful thing about community mobilisation?
  - If you could change something about the community mobilisation meetings, what would it be?

**IF NEVER PARTICIPATED IN ANY SISTERS ACTIVITIES: *Have you heard anything about Sisters with a Voice or CeSHHAR?***

- Who has told you about these services?
- What have they told you?
- Have you ever thought about attending the clinic or the meetings? Why or why not?
- How do you think these services are different to other health care in this area?

# Part IV: Health Behaviour

1. **Condom Supplies**

- Where can sex workers get condoms around here?
- Tell me about where you usually get condoms? [Probe: Do you get them from an Empowerment Worker or other sex workers? Do you get them from the Sisters clinic?]

1. **HIV Testing and services**

- Where can one get tested for HIV around here?
- Have you ever been tested? Where and how long ago?
- Tell me about your experiences with testing. [**Probe**: Have you ever had an HIV test at the Sisters clinic? How did you find the service?]

**ART Services**

- Where can sex workers get ARVs here?
- You don’t have to tell me any personal experiences if you don’t want to, but I would like to hear about sex workers’ experiences in general with taking ARV for HIV. What do you think about these services? [**Probe**: Tell me about HIV treatment at Sisters clinic]

What experiences have you had or heard about taking ARVs?

- Where can sex workers who have HIV get support? How do other sex workers treat them? [**Probe** about the following if not mentioned: stigma, social support, Empowerment Worker, SHG, Adherence Sisters]

**PrEP Services**

- Have you ever heard of PrEP? What can you tell me about it?
- You don’t have to tell me any personal experiences if you don’t want to, but I would like to hear about sex workers’ experiences in general with taking PrEP for prevention of HIV. What do you think about these services? [**Probe**: Tell me about PrEP at Sisters clinic]
- What experiences have you had or heard about taking PrEP?
- Where can sex workers who want to take PrEP get support? How do other sex workers treat them? [**Probe** about the following if not mentioned: stigma, fear of side effects, attitudes of peers and clients, Empowerment Worker, SHG, Adherence Sisters]

1. **Improvements**

- Are there changes to the Sisters programme that would make it easier for you and other sex workers to participate? Please describe. How can the project be better?

**Appendix B: AMETHIST Trial: Topic Guide for Empowerment Workers in Intervention Sites**

During this interview, the aim is to understand how Empowerment Workers/ Microplanners perceive their work as part of the AMETHIST trial, examining their perceptions of delivering all the components of MP, SHG and/or referrals, especially to the Sisters clinic. These interviews also explore the current social environment for sex workers, including their relationship with each other and the wider community, and to what EW perceive changes in social support networks and collective capacity among the sex workers they microplan/ manage in SHG.

# Part I: Background Information

1. **Can you tell me a little bit about yourself and why you became an Empowerment Worker?**

- How long have you been living and working in this area?
- Where do you work? Please describe your pattern of sex work, including where you find clients and how often you sell sex?
- How did you become involved with Sisters in general?
- Tell me about what motivated you to train as an EW?

1. **In this study, we are interested in what life is like for sex workers around here**

- Can you tell me about the challenges sex workers face?
- What are the positive aspects of being a sex worker here?
- In your experience, what do local sex workers feel is the most difficult part of working here?
- How do they cope with these kinds of difficulties? [**Probe**: whom can sex workers ask for help?]
- How do people in the community treat sex workers? [**Probe**: Local residents? Police? Health workers? Others?]

# Part II: Relationships between sex workers

1. **I would now like to ask you about how sex workers interact with each other. In your experience, how are relationships between the sex workers whom you know?**

- What different kinds of sex workers are there locally? [**PROBE**: Where do women sell sex? Do they have different types of clients?]
- How do different kinds of sex workers get along?
- Do you personally have close friendships with other sex workers?
- Can you give examples from work about how sex workers help each other out, either as part of work or outside of it? [**Probe**: Please give any personal examples you remember.]
- What about bad experiences? Can you describe any situations where other sex workers have caused problems for yourself or others whom you know? [**Probe**: Please give some examples.]
- Have relations among sex workers changed at all since you’ve been working here? Please describe any changes.

# Part III: Working for the Sisters with a Voice Programme

1. **Tell me about the training you attended to become a EW?**

- What did you like and what did you dislike about the training?
- Tell me some key topics you learned?
- Do you feel the training prepared you for your role as an EW? Why or why not? [**Probe**: In what way did you feel most and least prepared?]

1. **Please describe, step-by-step, what you did when you started working as an EW? [Ask the following probing questions only *after* the respondent has stopped describing her experiences in her own words)**

- *Hot Spot Mapping*: Tell me about this activity.
  - What did you find easy and difficult about the mapping process?
  - What kinds of challenges did you encounter during the mapping process?
  - Tell me about the mapping validation you did with an Outreach Worker?
- *Microplanning*: Please describe how you started this activity and what has happened until now?
  - Can you describe how you identified all the FSW in your hotspot?
  - How did FSW respond to being contacted by you?
  - Tell me about how you do the Risk Assessment, and what is easy or difficult about it.
  - How do you maintain contact with the FSW on your hotspot list? [**Probe**: What do you do when you can’t find someone or she has changed her phone number]
  - Do you think FSW like being microplanned? Why or why not?
  - What are the challenges of microplanning on a daily basis? [**Probe**: finding FSW, conducting risk-assessments, maintaining the monitoring forms, making clinic referrals]
  - What kinds of benefits do you think Microplanning has for FSW around here?
  - Are there negative aspects of microplanning for local FSW?
  - If you could change anything about microplanning what would it be?
- *Self-Help Groups* : If you have organised a Self-Help Group, please describe what you did and what is happening now.
  - How did you identify the members and bring them together?
  - Was it easy or difficult to motivate FSW to join a SHG?
  - What has helped establish a SHG?
  - What challenges have you faced?
  - How is the SHG going? [**PROBE**: what is going well, what problems are there?]
  - Can you describe any activities that are being planned by the SHG?
  - Do you think FSW like the SHG? Why or why not?
  - What are the challenges of leading a SHG on a daily basis? [**Probe**: attendance, conflicts, time constraints]
  - What kinds of benefits do you think SHG have for FSW around here?
  - Are there negative aspects of SHG for local FSW?
  - If you could change anything about how the SHG are organised, what would it be?
- Do you organise any *Community Mobilisation* sessions outside of the SHG?
  - **If YES**: How many times and what do you do? [**Probe**: frequency of activities, type of activities, levels of participation by FSW]
  - What do you think is the most useful thing about community mobilisation?
  - What do you think is not very useful about community mobilisation?
  - Do you think FSW like community mobilisation activities? Why or why not?
  - If you could change something about the community mobilisation meetings, what would it be?

# Part IV: Health Behaviour

1. **Condom Supplies**

- How do you distribute condoms to FSW?
- Do you find it easy or difficult to give out condoms? [**Probe**: Do you get them from and ORW or the clinic in time? Do you use the risk assessment to decide how many to distribute?]

1. **Referring FSW to the Sisters Clinic**

- Tell me about how you register local FSW at the Sister clinic.
- Tell me about your experiences with making referrals and encouraging FSW to attend the clinic. [**Probe**: Is this difficult or easy? Do many women refuse to go?]
- What do you think FSW like and don’t like about the Sisters clinic?
- Why do you think some FSW do not attend the clinic?
- How do you try to convince them?

**ART Services**

- How do you support FSW if you know they are on ART?
- What experiences do FSW around here have taking ARVs?
- What are the challenges with supporting FSW with HIV?
- What would make it easier for FSW with HIV to ensure they attend their appointments and take their ARV?

**PrEP Services**

- How do you support FSW if you know they are on PrEP?
- What experiences do FSW around here have taking PrEP?
- Why do you think some eligible women not want to start PrEP?
- What would make it easier for FSW to ensure they get tested regularly, attend their appointments and take PrEP?

1. **Supervision**

- Tell me about the Outreach Workers with whom you work? [**Probe**: how many come to this area to meet with you?]
- How often do you meet with an Outreach Workers from CeSHHAR?
- Tell me about these meetings. [**Probe**: how long do they last; what do you discuss?]
- What do you feel is useful and NOT useful about these meetings?
- How well do you feel supported by the Sisters programme?
- If you could change something about the way you are supervised by CeSHHAR, what would it be and why?

1. **Improvements**

- Are there changes to the Sisters programme that would make it easier for sex workers to participate? Please describe. How can the project be better?

**Appendix C: AMETHIST Trial: Discussion Guide for SHG in Intervention**

Self-Help Groups are already working as a collective unit, so the aim of the discussion is to understand the evolving dynamics and actions of SHG, including whether and how they build social support and cohesion among members and more broadly in the community. Discussions explore how SHG members came together, what motivated (and continues to motivate them) to meet, and what activities they have undertaken and plan in future. Discussions will also explore relationships between group members and their interactions with other sex workers and members of the local community.

# Part I: Background Information

1. **Can you tell me about how your SHG started?**

- How well did you know each other before?
- How was each member identified and contacted?
- Have you had the same members from the beginning or have there been some changes?
- What was the role of the Empowerment Worker?

1. **I’d like to hear about what motivated you as individuals to join a SHG?**

- What benefits did you expect from joining a SHG?
- Did any of you need to be encouraged or was everyone equally interested and motivated from the start?
- Are there other kinds of groups in the area that sex workers join? [**Probe**: Makando or other revolving or saving funds]
- What kinds of activities did you hope the SHG would conduct? Part II: Relationships between sex workers

1. **I would now like to ask you about how sex workers interact with each other. In your experience, how are relationships between the sex workers whom you know?**

- What different kinds of sex workers are there locally? [**PROBE**: Where do women sell sex? Do sex workers have different types of clients?]
- How do different kinds of sex workers get along? [**Probe**: would you say that FSW are friendly and supportive of each other? Why or why not?]
- What were the challenges of getting together in this group?
- How have other sex workers who know about your group reacted?

1. **So now can you tell me, step by step, what your SHG has been doing? [Ask probing questions only if the group runs out of things to say]**

- How often do you meet and where?
- Do you all come most of the time or are some members more active than others?
- How do you organise and conduct your meetings?
- How long do your meetings usually last?
- What activities have you done so far? [**Probe**: Have you been using the manual or developing your own topics?]
- What is going well?
- What is going less well?
- Do you have activities planned for the future?

1. **How does your SHG relate to other Sisters services around here?**

- Tell me about your relationship with your *Empowerment Worker* who contacts you? Do you see her outside of the SHG meetings, out in the community?
  - **If YES**: What do you talk about? What do you think about these meetings? How do you benefit? Are there any things you don’t like about being contacted by the Empowerment Worker?
  - **If NO**: Why not?
- Have you ever gone to the *Sisters clinic*?
  - **If YES**: Tell me about your experiences at the Sisters clinic. How do you feel about the treatment you get there? What are good and bad things about the clinic and the services there? How does the Sisters clinic compare to other health services around here?
  - **If NO**: Do you know about the Sisters clinic, which is a special service that come to the xxx-health centre ever xxx weeks to provide medical services and advice to sex workers? What do you think about it? Can you explain why you have never attended?

1. **Aspirations for the SHG**

- Now that your SHG has been running for a while, do you feel the levels of motivation and energy in the group are less, more or about the same as before? Please give examples.
- In addition to meeting regularly, are there other activities you want to do together?
- How easy or difficult do you think it is for the SHG to conduct events or activities on its own?
- What do you think about setting up a revolving savings fund, or working together on income-generating projects?
- What kind of contact do you have with other FSW in the community as a group?
- What kind of contact do you have with other community members as a group? [**Probe**: residents, police, religious leaders, other kinds of social groups etc]

1. **Health issues and services**

- Do you discuss health issues within the group? [**Probe**: What topics?]
- Do you ever accompany each other to a clinic?
- How can SHG members support each other and other sex workers to get the health care they need?

1. **Supervision**

- Tell me about the Empowerment Worker who helped set up this group.
- Does she attend all the group meetings?
- What kind of support do you want from her?
- Do you ever meet with one of the Outreach Workers from CeSHHAR? [**Probe**: how often, what do outreach workers do?]
- What do you feel is useful and NOT useful about these meetings?

1. **Improvements**

- Are there changes to the Sisters programme that would make it easier for you and other sex workers to participate? Please describe. How can the project be better?
